# Is preoperative anxiety associated with postoperative delirium in older persons undergoing cardiac surgery? Secondary data analysis of a randomized controlled trial

**DOI:** 10.1186/s12877-020-01872-6

**Published:** 2020-11-18

**Authors:** Koen Milisen, Bastiaan Van Grootven, Wim Hermans, Karen Mouton, Layth Al Tmimi, Steffen Rex, Elke Detroyer

**Affiliations:** 1 Department of Public Health and Primary Care, Academic Centre for Nursing and Midwifery, KU Leuven - University of Leuven, Kapucijnenvoer 35/4, B-3000 Leuven, Belgium; 2 Department of Geriatric Medicine, KU Leuven - University Hospitals Leuven, Herestraat 49, B-3000 Leuven, Belgium; 3 Research Foundation Flanders, Brussels, Belgium; 4 Department of Anesthesiology, KU Leuven - University of Leuven, University Hospitals of Leuven, Herestraat 49, B-3000 Leuven, Belgium; 5 Department of Cardiovascular Sciences, KU Leuven - University of Leuven, B-3000 Leuven, Belgium

**Keywords:** Aged, Anxiety, Delirium, Cardiac surgery, Risk factors

## Abstract

**Background:**

Although many studies have reported numerous risk factors for postoperative delirium, data are scarce about preoperative anxiety as a risk factor. The study aimed to investigate the association between preoperative anxiety and postoperative delirium in older patients undergoing cardiac surgery.

**Methods:**

Secondary data analysis of a randomized, observer-blind, controlled trial. A total of 190 patients 65 years or older and admitted to the intensive care unit and cardiac surgery unit of a university hospital scheduled for elective on-pump cardiac surgery were included. State anxiety was measured preoperatively using the Amsterdam Preoperative Anxiety and Information Scale and the Visual Analogue Scale for anxiety. Incidence of delirium was measured during the first 5 postoperative days using the Confusion Assessment Method for Intensive Care Unit (when ventilated), or the 3 Minute Diagnostic Interview for Confusion Assessment Method (when extubated) and by daily chart review.

**Results:**

Preoperative state anxiety was reported by 31% of the patients and 41% had postoperative delirium. A multiple step logistic regression analyses revealed no association between preoperative anxiety and postoperative delirium. Significant risk factors for postoperative delirium were age (OR = 1.10, 95% CI (1.03–1.18)), activities of daily living (0.69, 95% CI (0.50–0.96)), diabetes mellitus (OR = 3.15, 95% CI (1.42–7.00)) and time on cardiopulmonary bypass (OR = 1.01, 95% CI (1.00 to 1.02)).

**Conclusions:**

No relationship could be found between preoperative anxiety and postoperative delirium.

## Background

Delirium is a common neuropsychiatric condition in older patients characterized by a disturbance in attention and awareness, together with a disturbance in cognition or perception [1, 2]. The disturbance develops over a short period of time (usually hours to days) and tends to fluctuate in severity during the day [1, 2]. Delirium occurs in 3 to 52% of patients undergoing cardiac surgery [3, 4]. In particular, older surgical patients are at high risk [1].

Delirium has been associated with various adverse health outcomes, including cognitive and functional decline, higher morbidity and mortality, increased intensive care unit and hospital length of stay, and higher risk for institutionalization; and consequently leading to additional healthcare costs [1, 5].

Delirium is a multifactorial syndrome for which several predisposing factors have been suggested such as older age, pre-morbid depression and cognitive impairment, history of stroke and diabetes mellitus. In addition, several precipitating risk factors are involved including non-surgical (e.g. electrolyte disturbances, myocardial and pulmonary disorders, reduced sensory input, medication related problems such as newly initiated medication, increased doses, interactions etc.), as well as surgical risk factors (e.g. type and complexity of surgery, operative and anesthesia factors and postoperative complications such as pain, hypoxemia and infection) [3–5].

Although numerous studies have investigated these risk factors, less knowledge is available about preoperative anxiety as a risk factor for postoperative delirium. Notably, high levels of anxiety are reported by patients in the preoperative phase (21 to 77%) because of uncertainty about pain, the surgery and the surgical outcomes [6–9]. To the best of our knowledge, four studies investigated the relation of preoperative anxiety and postoperative delirium in older patients undergoing non-cardiac surgery [10–13] and only one study addressed this issue in patients undergoing cardiac surgery [14]. The majority of these studies could not show a significant association between preoperative anxiety and postoperative delirium. In only one recent study, with a limited sample size including older cancer patients, preoperative anxiety predicted postoperative delirium [13]. Although the pathophysiology of delirium is not fully understood, anxiety may induce aberrant stress responses (e.g. enhanced levels of circulating cortisol) and disturbances in neurohumoral mechanisms (e.g. microglial activation and disruptions of inflammatory cytokines, causing neuroinflammation), which all may contribute to the development of delirium [13].

Preoperative anxiety is a difficult to measure concept which might be an important reason for the failure of most former studies in older patient populations to show a relationship with postoperative delirium. Preoperative anxiety is usually defined as “state anxiety (e.g., situational anxiety) symptoms, reflecting a temporal and transient emotional state with changing intensity as a reaction to environmental stimuli” [14]. In all previous studies showing no relationship with postoperative delirium [10–12, 14], preoperative anxiety was measured by the State-Trait Anxiety Inventory (STAI) [15]. This instrument evaluates how people “generally feel” (by the Trait-Anxiety subscale) and how respondents feel “right now, at this moment” (by the State-Anxiety subscale) [15]. Although the State-Anxiety subscale has been recommended to evaluate preoperative anxiety, the questions in this instrument are not designed to measure the specific pre-operative context the patients are confronted with [16]. Therefore, other and newer measurement instruments have been suggested to better evaluate preoperative anxiety in the specific preoperative setup (e.g. the Amsterdam Preoperative Anxiety and Information Scale (APAIS)) [16, 17], and as a consequence warrant further investigation of its relationship with delirium.

If there might be a true relationship between preoperative anxiety and postoperative delirium, as was shown in only one study, it would strengthen preventive opportunities [13]. Indeed, non-pharmacological anxiety-reducing interventions such as preoperative music therapy and education could be further developed, tested and integrated into multicomponent strategies for the prevention of delirium [18].

Given the conceptual issues with regard to the evaluation of preoperative anxiety and the inconclusive results in the aforementioned studies, this study aimed to investigate the relationship between preoperative anxiety and the development of postoperative delirium among older persons undergoing cardiac surgery.

## Methods

### Design and sample

This secondary data analysis comprises data from a randomized, observer-blind, controlled trial (RCT), comparing the effect of xenon versus sevoflurane anesthesia on the incidence of postoperative delirium [19]. The primary study included 190 patients scheduled for elective cardiac surgery with the use of cardiopulmonary bypass (CPB) in the University Hospitals of the KU Leuven (Belgium) between November 2015 and December 2017. Patients were aged 65 years or older and able to provide written informed consent. Patients with the following characteristics were excluded: Dutch language barrier, chronic obstructive pulmonary disease GOLD > II, disabling neuropsychiatric illness such as dementia, schizophrenia, epilepsy or mental retardation. Patients with a recent history of drug or alcohol abuse, signs or symptoms of increased intracranial pressure, history of cerebrovascular accident or traumatic brain injury with residuals, risk factors/history for malignant hyperthermia were also excluded. Furthermore, patients were not eligible for the current study when known to have allergy to study medications and to have delirium at baseline. Patients in a critical perioperative state or requiring single lung ventilation were also excluded.

### Anesthesia and surgical intervention

General anaesthesia was induced with a continuous infusion of remifentanil 0.5 μg/kg/min followed by an IV bolus of propofol 0.5–1 mg/kg. After endotracheal intubation and ventilation, the randomization envelopes were opened and all patients were randomly allocated to an intervention or control group. The intervention group received xenon (40–60% in oxygen) anesthesia while sevoflurane (1.0–1.4%) was used in the control group [19]. In all patients, concentrations of anesthesia were cautiously titrated based on clinical signs for adequate depth of anesthesia, such as tachycardia, sweating, arterial hypertension, and patients’ movement and the use of an anesthesia depth monitor (bispectral index (BIS)) to achieve a BIS reading between 40 and 60. Furthermore, cardiorespiratory monitoring was performed based on our institutional clinical routine. All patients were planned for elective cardiac surgery on CPB. Based on the decision of the responsible cardiac surgeon, a normothermic or mild-moderate hypothermic CPB was used.

### Variables and measurement methods

#### Postoperative delirium

Incidence of postoperative delirium during the first 5 postoperative days was measured daily using the 3-min Diagnostic Confusion Assessment Method, a 20-item scale (10 cognitive testing items, 10 interviewer observations) based on the 4 core diagnostic features of the Confusion Assessment Method (CAM) algorithm [20, 21]. The Confusion Assessment Method for the Intensive Care Unit (CAM-ICU) was used to determine delirium in mechanically ventilated patients or when the 3D-CAM was not assessable [22]. In addition, a daily chart review was performed for the scores of the Intensive Care Delirium Screening Checklist (ICDSC) over the past 24 h, which was conducted by the bedside ICU nurse [23]. Patients who were deeply sedated and had a Richmond Agitation-Sedation Scale of ≤4 were not evaluable for postoperative delirium [22]. In the ward, a daily chart review for the results of the DOS (Delirium Observation Scale) over the previous 24 h was performed by the nurses [24]. Moreover, patients’ records over the previous 24-h period were checked to identify key words suggestive of postoperative delirium (e.g., “confused”, “aggressive”, “disorientated”, “agitated”, “drowsiness” and “delirious”) and the administration of anti-psychotic therapy [25]. All research nurses had received a specific training prior to the initiation of the study based on the 3D-CAM Training Manual for Research [21].

#### Preoperative anxiety

Preoperative anxiety was measured by two instruments. First, the Amsterdam Preoperative Anxiety and Information Scale (APAIS) is a six-item questionnaire measuring anxiety and the need for information with the use of 2 subscales on a five-point Likert scale ranging from 1 (“not at all”) to 5 (“extremely”) [16, 17]. Four items measure the anxiety for anesthesia and the surgical procedure (APAIS-A subscale) with a total score varying between 4 (not anxious) and 20 (highly anxious). A cut-off score of 11 or more is recommended to indicate preoperative anxiety [16] Since it is well-known that there is a positive association between anxiety and information requirements with regard to surgical and anesthetic procedures, two items measure the need for information (APAIS-NFI subscale) with a total score varying between 2 (no need for information) and 10 (high need for information). Patients with a total score of 5 or more are considered as having a moderate to high information requirement [16] Second, the Visual Analogue Scale for anxiety (VAS-A) was used to assess anxiety for the anesthesia and anxiety for the surgery, separately [26, 27]. Patients were asked to indicate their anxiety level on a scale between 0 (no anxiety) and 100 (worst possible anxiety).

### Demographics and other clinical data

The demographic data collected using chart review and preoperative patient interview were age, gender, level of education and living situation. We created a variable (e.g. treatment allocation) to classify patients according to the treatment to which they were randomized in the original clinical trial.

The following clinical data were collected based on the current evidence on potential risk factors of delirium in older patients undergoing cardiac surgery [3, 4, 28].

Pre-operative cognitive functioning was measured using the Mini-Mental State Examination (MMSE), a 30-item scale including several cognitive subdomains: orientation, calculation and attention, language, registration recall, and visual construction. The total score varies between 0 and 30, with a low score indicating cognitive dysfunction [29].

Pre-morbid functional status was evaluated with the Katz Index of activities of daily living (ADL), a scale that measures the (in)dependence regarding 6 activities of daily life (e.g. washing, dressing, toileting, moving, continence, feeding). Total scores vary between 0 and 12, with higher scores indicating a higher level of dependence [30].

The 10-item Geriatric Depression Scale (GDS) was used to assess the risk for depression pre-operatively. The total score ranges from 0 to 10, with higher scores indicating higher risk for depression [31].

Furthermore, serum levels of 25-hydroxy vitamin D (25-OHD) (nmol/l), diabetes mellitus, alcohol use (yes versus no or former), smoking (yes versus no or former), and the level of surgical risk assessed using the European System for Cardiac Operative Risk Evaluation II (EuroSCORE II) [32] were preoperatively determined.

Baseline and preoperative data (including pre-operative anxiety) were measured the day before surgery by trained research nurses.

The following intraoperative risk factors were collected: type of cardiac surgery, duration of anesthesia (minutes), total opioid dose (morphine and remifentanil) (μg), total sedation dose (mg), time on cardiopulmonary bypass (minutes), red blood cell transfusion (milliliters), highest intraoperative temperature (°C), mean arterial pressure (mmHg), and use of inotropic medication (yes versus no).

Finally, length of stay in the intensive care unit (minutes), and highest urea value (mg/dL) and lowest hemoglobin value (g/dL) preoperative until the 1st postoperative day and infection (presence of urinary tract infection, pneumonia, wound infection or sepsis) preoperatively until the 5th postoperative day were gathered.

### Ethical aspects

The study protocol was approved by the medical ethics committee of the University Hospitals Leuven (SR12/2014, version 2, 4th May 2015) and by the Federal Agency for Medicines and Health Products, Brussels, Belgium (16th, April 2015).

### Statistical analyses

Sample characteristics were explored using descriptive statistics. The mean and standard deviation were used for continuous data with a normal distribution, and the median and interquartile range (IQR) were used for continuous data that were skewed. Frequencies and percentages were used for categorical data.

The association between preoperative anxiety, demographical data, clinical data and postoperative delirium was first measured using univariable logistic regression. In a second step, a multivariable logistic regression model was build [33]. First, five key confounders were selected based on the evidence from previous studies (age, pre-operative Mini-Mental State Examination, Geriatric Depression Scale, Katz ADL index and EuroSCORE 2), and were added to the model. Treatment allocation was added as a confounder in the model to capture potential differences between the groups. Second, other predictors that were significant in the univariable analyses were added to the five key confounders in a stepwise manner using the Akaike Information Criteria (AIC) as selection criteria. The predictors were retained in the stepwise selection if the AIC remained the same or decreased (an increase would indicate a worse fit of the model). The *p*-values for the final selection of the model were ignored because selection based on p-values can result in optimistic estimates and overfitting. It has been demonstrated that the use of AIC in model selection did not result in optimistic estimates of the predictors. The five key confounders and the predictors retained based on the AIC constituted the main model. Third, the four anxiety predictors were tested in four separate models (one for each predictor). The statistical significance was determined using Odds Ratio’s (OR) 95% confidence intervals (95% CI). As a last step, we evaluated wheter adding an anxiety predictor to the main model increased the overall model performance (Δ Pseudo R2) discrimination (Δ C-index, Δ discrimination slope), and evaluated the fit of the model (Goodness-of-fit (GOF), chi2 test). Multicollinearity was evaluated using the Variance Inflation Factor (VFI). The analyses were performed using STATA/IC 15.

## Results

### Baseline characteristics

A total of 190 patients were included in the analysis. The mean age of the sample was 76 years old. On average, most patients demonstrated good premorbid cognitive and functional status. More details can be found in Table 1.

**Table 1:** Demographic and baseline characteristics

	Sample ***n*** = 190	Delirious ***n*** = 78	Non-delirous ***n*** = 112	OR (95% CI)
Age, mean (SD)	75.7 (5.9)	77.4 (5.8)	74.5 (5.6)	1.09 (1.04–1.15)
Male gender, *n* (%)	99 (52.1)	43 (55)	56 (50)	1.22 (0.69–2.19)
Living situation, *n* (%)				
Alone	50 (26.3)	21 (26.9)	29 (25.9)	1.05 (0.55–2.03)
Together	140 (73.7)	57 (73.1)	83 (74.1)	0.95 (0.49–1.83)
Educational level, *n* (%)				
< age 15	78 (41.3)	35 (44.9)	43 (38.7)	1.95 (0.82–4.63)
Age 16–18	77 (40.7)	33 (42.3)	44 (39.6)	0.92 (0.49–1.74)
College or university	34 (18.0)	10 (12.8)	24 (21.6)	0.51 (0.22–1.21)
Treatment allocation, *n* (%)				
Intervention	94 (49.5)	37 (47.4)	57 (50.9)	0.87 (0.49–1.55)
Anxiety measures				
APAIS-A score (4–20), mean (SD)	9.1 (3.8)	8.9 (43.8)	9.2 (3.8)	0.98 (0.91–1.06)
APAIS-A score ≥ 11, *n* (%)	59 (31.1)	21 (26.9)	38 (33.9)	0.72 (0.38–1.35)
APAIS-NFI (2–10), mean (SD)	5.4 (2.5)	5.0 (2.4)	5.6 (2.5)	0.91 (0.81–1.03)
APAIS-NFI score ≥ 5, *n* (%)	111 (58.4)	42 (53.9)	69 (61.6)	0.73 (0.40–1.31)
VAS-A for Anaesthesia (0–100), median (IQR)	24 (39)	25 (42)	22 (39.5)	1.00 (0.99–1.02)
VAS-A for Surgery (0–100), median (IQR)	37 (42)	37.5 (43)	37 (42)	0.99 (0.98–1.01)
Pre-operative Mini-Mental State Examination score (0–30), mean (SD)	27.8 (2.1)	27.5 (1.9)	28.0 (2.2)	0.88 (0.77–1.01)
Geriatric Depression Scale (0–10), median (IQR)	1 (2)	1 (2)	1 (2)	1.05 (0.88–1.26)
Katz ADL index (0–12), median (IQR)	0 (0)	0 (0)	0 (0)	0.95 (0.73–1.25)
Smoking status (Yes), *n* (%)	12 (6.3)	4 (5.1)	8 (7.1)	1.13 (0.83–1.52)
Drinking status (Yes), *n* (%)	70 (36.8)	23 (29.5)	47 (42.0)	0.68 (0.40–1.16)
Diabetes mellitus (Yes), *n* (%)	48 (25.3)	26 (33.3)	22 (19.6)	2.05 (1.05–3.97)
25-hydroxy vitamin D (25-OHD) (nmol/l), median (IQR)	22.4 (15.2)	20.7 (12.6)	24.2 (18.9)	0.98 (0.92–1.04)

Legend: *APAIS* Amsterdam Preoperative Anxiety and Information Scale, *APAIS-A* Anxiety subscale of APAIS scale, *APAIS-NFI* need for information subscale of APAIS scale, *VAS-A* Visual Analogue Scale for anxiety, *ADL* activities of daily living, *IQR* interquartile range

### Incidence of preoperative anxiety and need for information

A total of 59 patients (31%) had state anxiety symptoms prior to surgery according to the APAIS-A subscale. A total of 111 (58%) patients had moderate to high information requirements according to the APAIS-NFI subscale (see Table 1).

### Incidence and risk factors for delirium

A total of 78 patients (41%) experienced delirium.

Compared to non-delirious patients, delirious patients were significantly older, were more likely to have diabetes mellitus, had higher levels of surgical risk (i.e., higher EuroSCORE II), had longer duration of cardiopulmonary bypass and duration of anesthesia, longer lengths of stay in the intensive care unit and higher rates of postoperative infections (see Tables 1 and 2).

**Table 2:** Surgical characteristics

	Sample ***n*** = 190	Delirious ***n*** = 78	Non-delirous ***n*** = 112	OR (95% CI)
EuroSCORE II, median (IQR)	3.9 (5.3)	6.1 (5.8)	3.3 (4.3)	1.13 (1.06–1.21)
Type of surgery *n* (%)				
Valve	185 (97.4)	75 (96.2)	110 (98.2)	0.45 (0.07–2.79)
CABG	75 (39.5)	37 (47.4)	38 (33.9)	1.75 (0.97–3.18)
Combined surgery, *n* (%)	72 (37.9)	35 (44.8)	37 (33.0)	1.65 (0.91–2.99)
Time on cardiopulmonary bypass (min), mean (SD)	120.0 (43.0)	130.4 (48.0)	112.6 (37.9)	1.01 (1.00–1.02)
Duration of anaesthesia (min), mean (SD)	283.0 (75.5)	303.3 (83.7)	268.7 (65.8)	1.01 (1.00–1.01)
Total morphine dose (μg) intraoperative, mean (SD)	4385.1 (1546.0)	4626.2 (1677.6)	4220.0 (1433.6)	1.01 (1.00–1.01)
Total sedation dose (mg) intraoperative, mean (SD)	878.2 (335.7)	901.9 (371.1)	861.8 (309.4)	1.00 (0.99–1.01)
Red blood cell transfusion (ml) intraoperative, median (IQR)	254 (525)	272.5 (565)	248 (520.5)	1.00 (0.99–1.01)
Mean cardiac output (l/min) intraoperative, mean (SD)	4.2 (1.5)	4.4 (2.0)	4.2 (0.9)	1.10 (0.88–1.36)
Use of inotropic medication (Yes) intraoperative, *n* (%)	108 (56.8)	47 (60.3)	61 (54.5)	1.27 (0.71–2.28)
Highest intraoperative temperature (°C), mean (SD)	36.6 (0.4)	36.5 (0.4)	36.6 (0.4)	0.76 (0.37–1.54)
Mean arterial pressure (mm Hg) intraoperative, mean (SD)	75.0 (9.2)	74.9 (9.2)	75.0 (9.3)	0.99 (0.97–1.03)
Lowest haemoglobin value (g/dL) intraoperative, mean (SD)	9.5 (1.0)	9.4 (1.1)	9.6 (0.9)	0.82 (0.61–1.10)
Highest urea value (mg/dL) interoperative, median (IQR)	36 (17)	38 (19)	35 (15)	1.01 (0.99–1.02)
Length of stay in the intensive care unit (hours), median (IQR)	48.1 (68.7)	49.9 (68.6)	47.4 (69)	1.0 (0.99–1.01)
Postoperative infection (Yes), *n* (%)	43 (22.6)	25 (32.1)	18 (16.1)	2.46 (1.23–4.93)

Legend: *EuroSCORE II* European System for Cardiac Operative Risk Evaluation II, *CABG* Coronary Artery Bypass Grafting, *IQR* interquartile range

The final main model included 8 predictors: age, pre-operative cognitive functioning, depression preoperatively, premorbid activities of daily living, surgical risk, duration of cardiopulmonary bypass, diabetes mellitus and infection. Significant risk factors for delirium were age (OR = 1.10, 95% CI (1.03–1.18)), activities of daily living (0.69, 95% CI (0.51–0.96)), diabetes mellitus (OR = 3.15, 95% CI (1.42–7.00)) and time on cardiopulmonary bypass (OR = 1.01, 95% CI (1.00 to 1.02)) (see Table 3). There was no association between anxiety measures and delirium onset in both the univariate and the multivariate main model. The model performance also did not improve when adding the anxiety predictors to the main model (Δ R2 = 0, Δ C-index = 0, Δ Discrimination slope = 0) for each of the four predictors in comparison with the main model).

**Table 3:** Risk factors for delirium

Risk factor	Unadjusted OR (95% CI)	Adjusted OR (95% CI)
**Main model** ^a^		
Treatment allocation	0.87 (0.49–1.55)	1.19 (0.61–2.32)
Age	1.09 (1.04–1.15)	1.10 (1.03–1.18) *
Mini-Mental State Examination	0.88 (0.77–1.01)	0.90 (0.77–1.06)
Geriatric Depression Scale	1.05 (0.88–1.26)	1.04 (0.84–1.29)
Katz ADL index	0.95 (0.73–1.25)	0.69 (0.50–0.96) *
EuroSCORE 2	1.13 (1.06–1.21)	1.07 (0.99–1.15)
Time on cardiopulmonary bypass	1.01 (1.00–1.02)	1.01 (1.00–1.02) *
Diabetes mellitus	2.05 (1.05–3.97)	3.15 (1.42–7.00) *
Postoperative infection	2.46 (1.23–4.93)	1.90 (0.87–4.12)
**Anxiety measures** ^b^		
APAIS, anxiety score ^c^	0.98 (0.91–1.06)	0.97 (0.88–1.07)
APAIS, need for information score ^d^	0.91 (0.81–1.03)	0.95 (0.83–1.10)
Visual Analogue Scale, anaesthesia ^e^	1.00 (0.99–1.02)	1.00 (0.99–1.02)
Visual Analogue Scale, surgery ^f^	0.99 (0.98–1.01)	1.00 (0.98–1.01)

* Statistical significant at *p* < 0.05

^a^ Performance of the main model: Pseudo R2 = 0.17, C-index = 0.77, Discrimination slope = 0.21 GOF chi2 = 34.98, *p* = 0.760, Mean VIF = 1.18;

^b^ Anxiety measures were tested seperately in four different models: model 1 = Main model + APAIS anxiety score, model 2 = Main model + APAIS need for information score, model 3 = Main model + Visual Analogue Scale anaesthesia, model 4 = Main model + Visual Analogue Scale surgery

^c^ Performance of Main model + APAIS anxiety score (in comparison with main model): Δ R2 = 0, Δ C-index = 0, Δ Discrimination slope = 0; GOF chi2 = 5.88, *p* = 0.661

^d^ Performance of Main model + APAIS need for information score (in comparison with main model): Δ R2 = 0, Δ C-index = 0, Δ Discrimination slope = 0; GOF chi2 = 5.94, *p* = 0.654

^e^ Performance of Main model + Visual Analogue Scale anaesthesia (in comparison with main model): Δ R2 = 0, Δ C-index = 0, Δ Discrimination slope = 0; GOF chi2 = 7.03, *p* = 0.534

^f^ Performance of Main model + Visual Analogue Scale surgery (in comparison with main model): Δ R2 = 0, Δ C-index = 0, Δ Discrimination slope = 0; GOF chi2 = 6.56, *p* = 0.585

*Abbreviations*: *APAIS* Amsterdam Preoperative Anxiety and Information Scale, *ADL* activities of daily living

## Discussion

Compared to the few studies investigating the relationship between preoperative anxiety and postoperative delirium [10–14], the current study is the first study to investigate this relation by using measurement scales (e.g. APAIS and VAS-A scale) specifically designed to evaluate state anxiety related to the specific situation the patient is confronted with (e.g. the surgical and anesthetic procedures).

In this study, the incidence of delirium was 41%; confirming that this is a common complication in older patients undergoing cardiac surgery, as shown in previous studies [3, 4]. Overall, 3 out of 10 patients had state anxiety symptoms preoperatively and almost 60% indicated moderate to high information requirements. The latter represents an important aspect in patients’ experiences of the preoperative phase as information given to the patients before surgery has been shown to facilitate recovery [16, 32]. Despite these high numbers of patients with anxiety and information needs, preoperative anxiety did not increase the odds of having postoperative delirium, which concurs with the results found in most of previous studies [10–12, 14].

The fact that a relationship between preoperative anxiety and postoperative delirium could not been demonstrated does not mean that interventions targeted to cope with anxiety preoperatively should be neglected. Indeed, excess preoperative anxiety may cause pathophysiological reactions such as tachycardia, hypertension and arrhythmias [17]. Furthermore, studies have shown associations between preoperative anxiety and other deleterious effects in the immediate and long-term postoperative period such as higher rates of complications and pain, less hemodynamic stability, increased use of analgesics and anesthetic needs, atrial fibrillation, longer hospital stay, increased risk of readmission, higher cardiovascular morbidity and mortality and poorer quality of life [27, 34–36].

Reasons, why a relationship between preoperative anxiety and postoperative delirium in this and previous studies [10–12, 14] could not be shown, remain speculative. Except for difficulties in operationalization and measurement of preoperative anxiety, the fact that most patients in this study demonstrated good premorbid cognitive and functional status might have contributed to this finding. Indeed, the risk for delirium is determined by the interaction of predisposing and precipitating factors; meaning that the lower the premorbid vulnerability of the patient (e.g. good premorbid cognitive and functional status), the more precipitating risk factors are necessary in order to develop delirium [37]. Perhaps, in patient populations with a lower frailty profile, non-psychological risk factors might play a more important role compared to psychological risk factors in this interaction, such as the risk factors found in the final main model of this study (e.g. age, ADL functional status, diabetes mellitus and the time on cardiopulmonary bypass). Since this is only speculative, more research is necessary to confirm this hypothesis.

Only very recently, a study in 91 cancer patients with a relatively young age (average age of 66 years old) could demonstrate for the first time a relationship between preoperative anxiety and postoperative delirium [13]. Although the authors could not clearly explain why preoperative anxiety predicted postoperative delirium, this study used the anxiety subscale of the Hospital Anxiety and Depression Scale (HADS-A) [38]; an instrument measuring “preexisting” anxiety symptoms occurring in the previous 2 weeks before the operation, which is conceptually different from preoperative anxiety measured “immediately” before the upcoming surgery (i.e., the day before the surgery).

The current study has the following strengths. First, the use of internationally validated and standardized instruments to diagnose delirium and preoperative anxiety. Second, a rigorous multiple step statistical approach testing the relationship between preoperative anxiety and postoperative delirium. And third numerous risk factors previously shown to be related with postoperative delirium in older patients with cardiac surgery were taken into account.

We acknowledge, however, that the present study also has some limitations. First, the study is a secondary data analysis of an intervention trial in which the testing of xenon could have biased our results. However, we think this is unlikely since the intervention trial did not show a statistical difference in the incidence of delirium between the intervention and control group [39]; and because we corrected for potential group differences. Second, the study included mainly older patients with good premorbid cognitive and functional status, limiting the generalizability of our results to older patients with a higher premorbid co-morbidity. Third, we could not clearly explain why no relation between preoperative anxiety and postoperative delirium was found, and clarifications aforementioned were only speculative warranting further research. Fourth, some of the confidence intervals around the estimates were wide. Readers should take caution inferring the exact association of the variables in the final model, and type-two errors can’t be excluded.

## Conclusions

The present study could not identify a relationship between preoperative anxiety and postoperative delirium; congruent with the results of most earlier studies [10–12, 14]. However, given the inconclusive results when compared to the results of a recent study showing preoperative anxiety as a predictor of postoperative delirium in cancer patients [13], further research is warranted. If a relationship indeed would exist, the further development of intervention strategies to reduce preoperative anxiety may gain importance for the prevention of postoperative delirium.

## Data Availability

The datasets used and/or analysed during the current study are available from the corresponding author on reasonable request.

## Abbreviations

STAI: State-Trait Anxiety Inventory
APAIS: Amsterdam Preoperative Anxiety and Information Scale
APAIS-A subscale: Anxiety subscale of Amsterdam Preoperative Anxiety and Information Scale
APAIS-NFI subscale: Need for Information subscale of Amsterdam Preoperative Anxiety and Information Scale
RCT: Randomized controlled trial
CPB: Cardiopulmonary Bypass
BIS: Bispectral Index
CAM: Confusion Assessment Method
CAM-ICU: Confusion Assessment Method for the Intensive Care Unit
ICDSC: Intensive Care Delirium Screening Checklist
DOS: Delirium Observation Scale
VAS-A: Visual Analogue Scale for anxiety
ADL: Activities of Daily Living
IQR: Interquartile Range
MMSE: Mini-Mental State Examination
GDS: Geriatric Depression Scale
25-OHD: 25-hydroxy vitamin D
EuroSCORE II: European System for Cardiac Operative Risk Evaluation II
GOF: Goodness-of-fit
OR: Odds Ratio’s
CI: Confidence intervals
VFI: Variance Inflation Factor
HADS-A: Anxiety subscale of the Hospital Anxiety and Depression Scale
